# Developments towards a Multiscale Meshless Rolling Simulation System

**DOI:** 10.3390/ma14154277

**Published:** 2021-07-30

**Authors:** Umut Hanoglu, Božidar Šarler

**Affiliations:** 1 Institute of Metals and Technology, Lepi Pot 11, 1000 Ljubljana, Slovenia; bozidar.sarler@fs.uni-lj.si; 2 Faculty of Mechanical Engineering, University of Ljubljana, Aškerčeva 6, 1000 Ljubljana, Slovenia

**Keywords:** meshless methods, radial basis functions, hot rolling, steel, recrystallization, austenite grain size, ferrite grain size

## Abstract

The purpose of the present paper is to predict the grain size of steel during the hot-rolling process. The basis represents a macroscopic simulation system that can cope with temperatures, stresses and strains of steel in a complete continuous rolling mill, including reversible pre-rolling and finishing rolling with several tenths of rolling passes. The grain size models, newly introduced in the present paper, are one-way coupled to the macro-scale calculations performed with the slice model assumption. Macroscale solution is based on a novel radial basis function collocation method. This numerical method is truly meshless by involving the space discretization in arbitrarily distributed nodes without meshing. A new efficient node generation algorithm is implemented in the present paper and demonstrated for irregular domains of the slice as they appear in different rolling passes. Multiple grain size prediction models are considered. Grain size prediction models are based on empirical relations. Austenite grain size at each rolling pass as well as the ferrite grain size at the end of rolling are predicted in this simulation. It is also shown that based on the rolling schedule, it is highly likely that recrystallization takes place at each pass throughout a continuous rolling mill. The simulation system is coded as a user-friendly computer application for industrial use based on programming language C# and an open source developer platform NET and runs on regular personal computers the computational time for a typical rolling simulation is usually less than one hour and can thus be straightforwardly used to optimize the rolling mill design in a reasonable time.

## 1. Introduction

**Figure 1:**
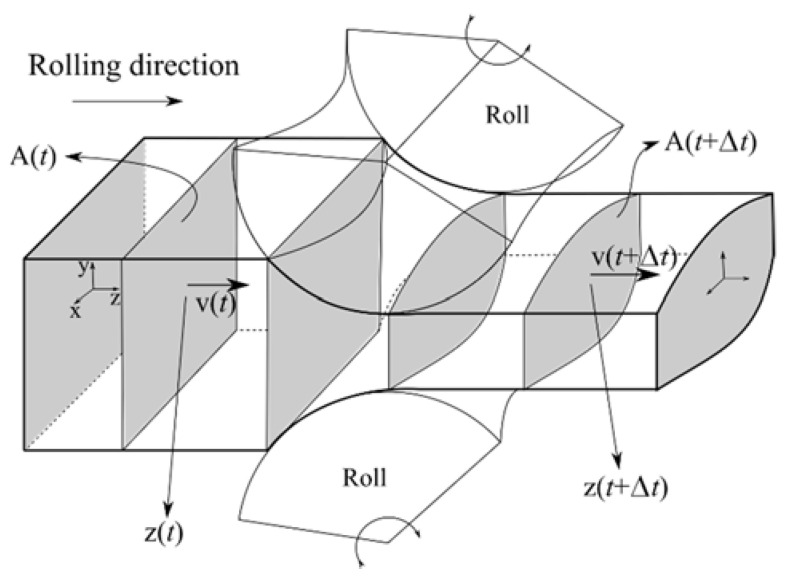
Scheme of slice model used in the macroscopic rolling simulations. A(t) is area, v(t) is velocity and z(t) is position.

In the present paper, the macroscopic simulation system, coping with a complete continuous rolling mill, including reversible pre-rolling and finishing rolling with several tenths of rolling passes [1,2,3,4,5], is upgraded to deal with grain size prediction. The main aim is to calculate, in addition to the temperature and deformation fields, the austenite grain size at each rolling pass and ferrite grain size at the end. The microscopic grain-size prediction models are one-way coupled to the macroscopic thermo-mechanical simulation. A previously developed 2D numerical model [6], based on the slices that allow only plane strain deformation and are aligned perpendicular to the rolling direction, is used. The slices on which 2D stresses and temperature fields are computed are illustrated in Figure 1.

Shaping any metal, such as in the hot rolling process, requires consideration of large plastic deformation. An overview of related multiscale physics is given in the following paragraphs. The start of this plastic deformation process is triggered when the dislocations can move inside a grain. This threshold is the yield stress, and its value depends on the dislocation density. By applying deformation and heat treatment, dislocation density and the grain sizes face a significant variation.

If the strain rate and temperature are high enough, a different phenomenon, dynamic recrystallization, occurs. The dynamic recrystallization depends on the critical strain. As a result, new grain sizes appear, either partially or fully. However, not all recrystallization takes place immediately after exceeding the critical strain. After a particular time, meta-dynamic crystallization may take place.

When the deformation is small, or there is no deformation, a softening process may occur due to sufficient time at high temperatures. The softening can again lead to partial or complete static recrystallization. Static recrystallization usually leads to larger grains with higher temperatures [7]. The stored energy in a material is proportional to the defect density. Therefore, an increase in grain size decreases the total grain boundary area and as a result, the stored energy is reduced.

The numerical prediction of grain size constitutes an essential aspect of microstructure studies [8]. Smaller grains may be desired since they lead to higher strength because it is harder for dislocations to pass over the grain boundaries during plastic deformation. Or the other way around for the ductility. The steel industry needs to predict the mechanical properties of their final product, which is only possible with the microstructure analysis [9].

In this paper, multiple grain size prediction models [10,11,12,13,14,15,16,17] were coupled with the macroscopic rolling simulation system. The basis of the micro-scale simulation used in the present paper is based on the model predicted by Hodgson and Gibbs [10] with minor adjustments. Multiple grain size prediction models for different types of steels were gathered. These models consist of empirical correlations to determine the critical strain and grain size after meta-dynamic, dynamic or static recrystallization. All the necessary input data for these models were obtained from the macroscopic 2D slice model results. Checking for possible recrystallization type and predicting the corresponding austenite grain size was performed immediately after obtaining the macroscopic results for each slice. After the hot rolling process was completed, the rolled steel was still hot and considered mainly in the austenite phase. Based on the models found in the literature, ferrite grain size prediction could be made as a function of the cooling process. Ferrite transformation temperature is calculated based on the material composition, and in the simulations, the steel is cooled down just below that transformation temperature. The discussion of how to implement the microscopic models can be found in textbooks [1,9]. Since it was not possible to obtain all the correlations for each steel grade separately for all the micro-scale simulation sections, a combination of those relations for various steel types were gathered and also applied. This was just to show that in this way, both macro and micro simulation results of hot rolling could be obtained easily by using a single meshless simulator. A specific model was used for each sub-section of micro simulation, but the necessary parameters were material dependent, and they could also be user-defined. In the results section, the first macro scale simulation results by using 16MnCrS steel were given. Later the same results were used as inputs for multiple micro-scale simulations. Comparison of different grain size prediction models were shown to distinguish the behaviour of each model. Each collocation point in a computational domain represents a small area with a uniform grain structure.

Over the years, a comprehensive rolling simulation system has been developed for industrial use. The supporting steel factory provides rolling schedules in use, and macro-scale simulations have been up to now run by a successful macroscopic hot rolling simulation system [18]. The meshless solution of the macro-scale deformation was chosen here since it is very straightforward to distribute collation nodes over a computational domain. Therefore, it was easy to implement numerically and also did not require any background integration. Meshless methods were multiple times proven to solve thermal [19] and mechanical [20] problems efficiently and accurately. Recently, micro-scale simulation capabilities based on empirical relations found in the literature were added, and a one-way coupling between macro and micro-scale models was formed. The motivation for the present research was understanding and simulating the material behaviour in between the passes, which is usually ignored during a multi-pass rolling. In the majority of the simulations, the initial material definition was used throughout the simulation regardless of the number of deformation steps, time and temperature. This work clearly shows the possibility of recrystallization at each pass separately during hot rolling and how much the material properties may change, based on the change of the grain size.

In the present paper, the possibility of complete or partial recrystallization is modelled. This makes more realistic the assumption of complete recrystallization at each roll pass, as assumed in some publications [17]. Furthermore, the effects of the chemical composition of the steel billet on its macro response and grain structure are also investigated.

## 2. Materials and Methods

**Figure 2:**
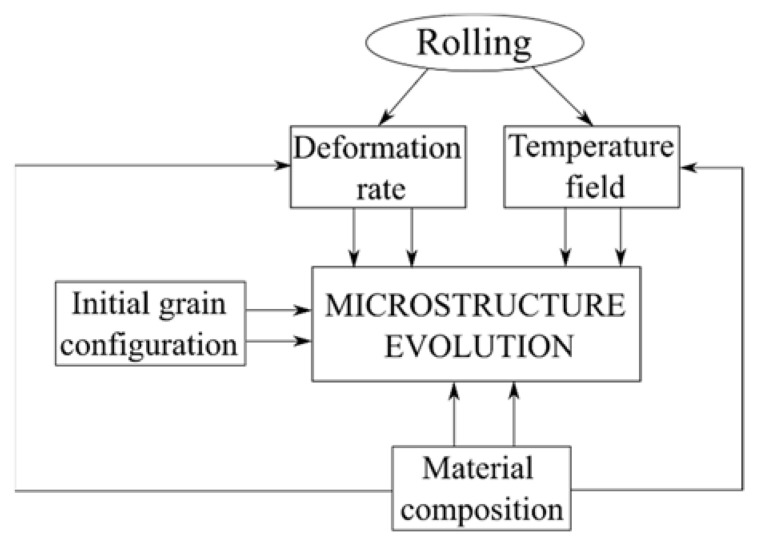
Picture of one-way macro-micro coupling.

It is pointed out by Sellars [8] that the experimental tests or industrial trials are not enough to understand all the details of the hot rolling process since it is impossible to control or monitor all the parameters of rolling in industrial conditions and even in laboratory conditions. Therefore, the best solution suggested by Sellars [14] is combining the numerical simulations with empirical models based on experiments. A macro-scale numerical prediction of the deformation of steel with heat transfer during hot rolling has been previously developed [6,18]. To achieve the microstructure evolution just as described in Figure 2, we need to know the initial grain configuration and empirical models that depend on the involved steel grade.

### 2.1. Macro Scale Solution

**Figure 3:**
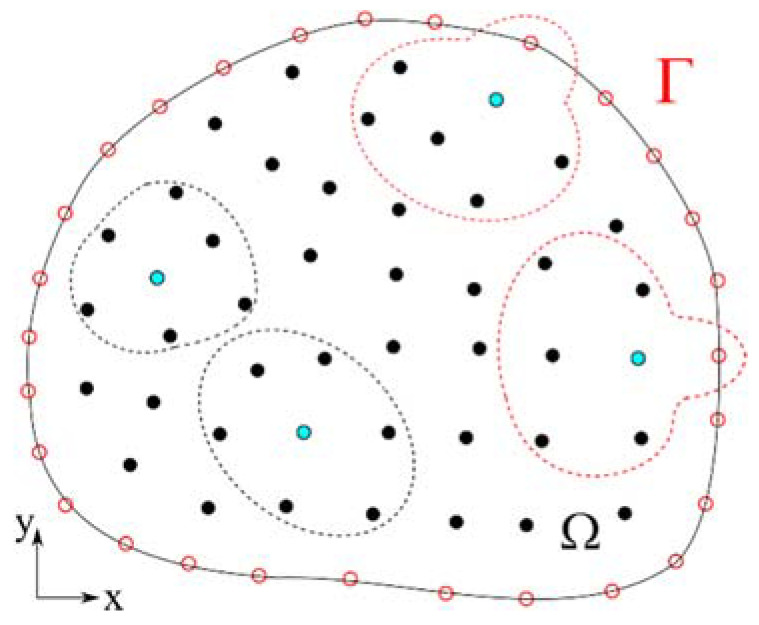
Scheme of the domain Ω and boundary Γ discretization with indicated local influence domains consisting of 7 node and for each central node in an influence domain is marked in blue.

Thermo-mechanical simulation of rolling is based on 2D slices aligned towards the rolling direction. The slices were simulated one after another, and both thermal and mechanical models were solved in a coupled way

(1) $$L^{T}\sigma +b=0,$$

(2) $$\rho c_{p}\frac{\partial T}{\partial t}=\nabla \cdot \left(k\nabla T\right)+\dot{Q},$$

where L,σ,b are derivative operator matrix, stress vector and body force vector, respectively. $\rho ,c_{p},T,k,\dot{Q}$ stand for the density, specific heat, temperature, thermal conductivity and internal heat generation, respectively. The meshless numerical procedure is based on the interpolation of an unknown field θ(p) at position $p=p_{x}i_{x}+p_{y}i_{y}$, interpolated with radial basis shape functions ψ(p) over seven neighbouring nodes (N) as shown in Figure 3,

(3) $$\theta \left(p\right)=\sum_{i=1}^{N}\psi_{i}\left(p\right)\alpha_{i},$$

where $\alpha_{i}$ are the collocation coefficients that need to be determined. The partial derivatives of the unknown field can be calculated as

(4) $$\frac{\partial \theta \left(p\right)}{\partial x_{j}}=\sum_{i=1}^{N}\frac{\partial \psi_{i}\left(p\right)}{\partial x_{j}}\alpha_{i}.$$

The temperature in the thermal model and the mechanical model’s displacement field are interpolated with the multi-quadric (MQ) radial basis functions (RBF). The details of the solution of these two models have been previously published in [21].

**Figure 4:**
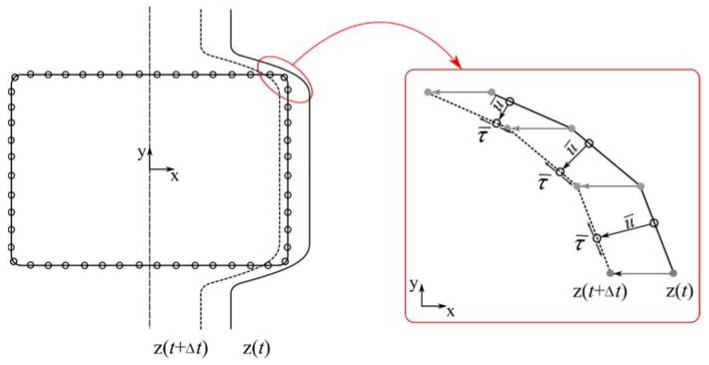
Boundary nodes during contact with groove surface and applied boundary conditions.

The boundary condition for the thermal model is of the Robin type,

(5) $$-k\frac{\partial T}{\partial n_{\Gamma }}=h\left(T-T_{}^{ref}\right).$$

where k is thermal conductivity, $n_{\Gamma }$ is unit normal vector on the surface, h is heat transfer coefficient, and $T^{ref}$ is the reference temperature. The majority of complications during simulation usually appear at the contact boundaries between the strand and the groove. The heat flux there becomes very high in comparison with the non-contact boundaries. At the contact boundaries, $T^{ref}$ is considered as the roll’s surface temperature. The boundary conditions for the mechanical model are described both with the prescribed traction ($\bar{\tau }$) and the prescribed displacement ($\bar{u}$)

(6) $$\bar{\tau }=-\mu p,\;\bar{u}=u,$$

where μ is the coefficient of friction, p is stress acting on the boundary, and u is the displacement. In some rare cases, when the groove surface has a unique geometry, prescribed traction boundary condition may not be appropriately satisfied, and might lead to erroneous results. To overcome this issue, during the calculation step at first contact, an artificially sticking boundary condition was applied to some of the boundary points. This resulted in no relative motion between the roll surface and the boundary in one calculation step. Later all the boundary nodes received proper boundary conditions defined in Equation (6) as schematically seen in Figure 4.

Interpolating an unknown field with regular node distribution over a rectangular domain works perfectly with meshless Local Radial basis Function Collocation Method (LRBFCM) and is also easy to implement numerically. However, severe deformation simulations, such as rolling or forging, require redistribution of the computational nodes. In other words, a solution with the regular node distribution becomes impossible in large deformations. An essential advantage of the LRBFCM is that it gives stable results also with scattered node distribution. A comparison of the results is shown later in this paper where a rectangular domain is interpolated with regular and scattered node distribution.

LRBFCM interpolates the temperature field in the thermal model, and the governing equation is solved through explicit time stepping. The time step is chosen as 1 ms. The nodes of the mechanical and thermal models coincide. The solution is obtained through local interpolation for each influence domain without creating a global matrix. In the mechanical model, interpolation of displacement filed over each local influence domain can be assembled into a global solution matrix (A) by replacing the local collocation coefficients with the inverse of the local interpolation matrix and the local displacement vector. Hence, the system of equations can be written in the following form

(7) AU=B,

and solved for global column matrix of displacements $U={\left[u_{x1},u_{y1},\ldots \ldots ,u_{xN},u_{yN}\right]}^{T}$ with the size of two times the number of collocation nodes (2N). The adjacent vector B includes the boundary values. As a result of the nonlinear material model of steel, the solution matrix A depends on the current state of displacements. The solution can only be achieved iteratively. In this paper, a direct iteration method was chosen due to its simplicity and to avoid assembling the Jacobian matrix. The local solution of the system of equations leads to a sparse matrix A, and with the help of the Intel math kernel library [22], it can be solved efficiently by direct iteration with iteration index j.

(8) $$A\left(U^{j}\right)U^{j+1}=B,$$

### 2.2. Micro Scale Solution

**Figure 5:**
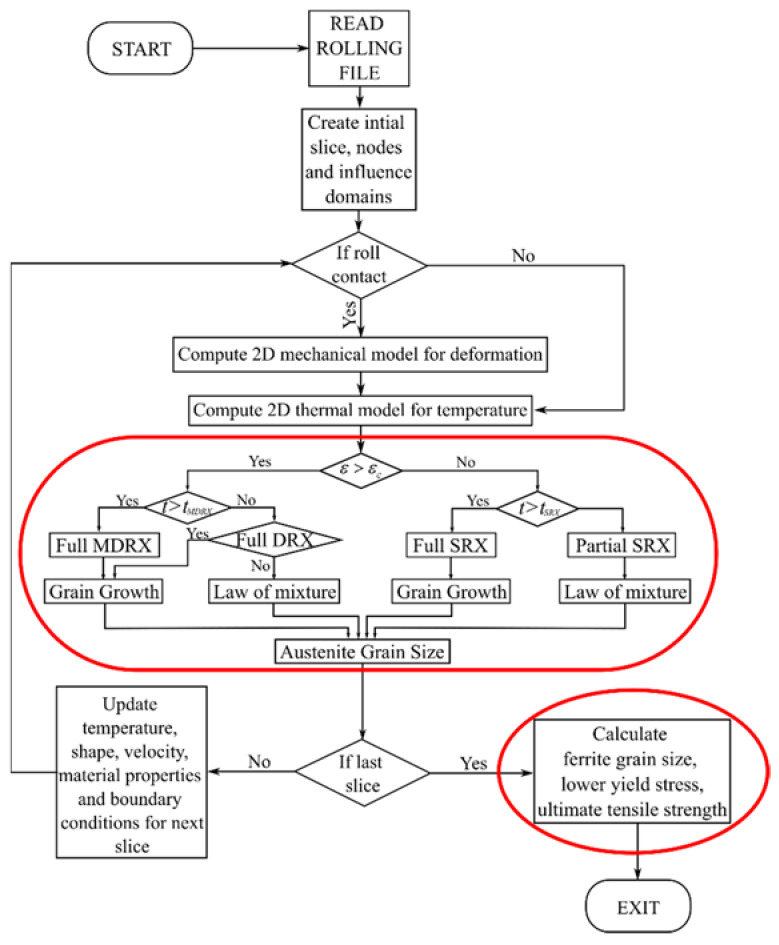
Scheme of the multiscale rolling simulation system. Micro-scale simulation steps are circled with red.

In the present work, the critical strain, the time for dynamic, meta-dynamic or static recrystallization and the grain sizes after different types of recrystallization implemented through a sequence of different empirical models. It is assumed that each collocation point in a computational domain represents a small area with a uniform grain structure. Grain size prediction models were obtained for the austenite phase. Solution steps were adopted from Hodgson and Gibbs [10], as shown in Figure 5.

#### 2.2.1. Critical Strain

The micro-scale modelling starts by checking first if the strain field in some of the points, some of the sub-areas or globally exceeds the critical strain value ($\varepsilon_{c}$)

(9) $$\varepsilon_{c}=A_{c}{\underset{Z}{\underbrace{\left(\dot{\varepsilon }\exp\frac{Q_{d}}{RT}\right)}}}^{p}D^{q},$$

where Z denotes the Zener–Hollomon parameter ($s^{-1}$), used in a multitude of empirical relations, $\dot{\varepsilon }$ is strain rate, $Q_{d}$ is activation energy (J/mol K), R is ideal gas constant 8.314 J/mol K, T is temperature in Kelvin and D is the initial austenite grain size diameter. The coefficients in Equation (9) for different steel grades are listed in Table 1. The dynamic or meta-dynamic recrystallization is triggered at the points where the critical strain value is exceeded. This can occur locally or on the global scale of the domain.

#### 2.2.2. Dynamic Recrystallization

The dynamic recrystallization (*DRX*) occurs when the critical strain is reached, but time is not long enough for meta-dynamic recrystallization. The grain size is predicted from the following equation

(10) $$D_{DRX}=A_{d}Z^{r},$$

The coefficients of Equation (10) are defined in Table 2 below.

#### 2.2.3. Meta-Dynamic Recrystallization

The meta-dynamic crystallization is also called post dynamic recrystallization. Its effect on the grain size is similar to the dynamic recrystallization; however, there is a time-based criterion that includes strain rate. In this paper, the following definition is used [15],

(11) $$t_{MDRX}=1.166\varepsilon^{-0.8},$$

as developed by Manohar et al. If the current time t at any deformation step exceeds the criterion ($t>t_{MDRX}$), it is considered that the meta-dynamic recrystallization (*MDRX*) occurs. If this is the case, the grain sizes must be recalculated from the following equation,

(12) $$D_{MDRX}=A_{m}Z^{r}.$$

The grain size prediction model for the meta-dynamic and dynamic recrystallization is the same, but the coefficients are different. The constants of Equation (12) are defined in Table 3 below.

#### 2.2.4. Static Recrystallization

There is a time criterion for static recrystallization (SRX) to occur, and in this paper, a model defined by Zhang et al. [16] for high carbon steel is used. It has the following form,

(13) $$t_{SRX}=1.944\times 10^{-4}\varepsilon^{-1.0}D_{0}^{0.6}{\left(\frac{3.6}{\dot{\varepsilon }}\right)}^{0.28}\exp\left(\frac{6900}{RT}\right).$$

when the time during the simulation is larger than $\left(t>t_{SRX}\right)$ then, static recrystallization may be expected. The grain size after the recrystallization is obtained from

(14) $$D_{SRX}=A_{s}\varepsilon^{m}D_{0}^{r}$$

The coefficients for Equation (14) are defined in Table 4.

#### 2.2.5. Grain Growth

After complete recrystallization, the grains will grow until the next partial or complete recrystallization. In the present paper, the following grain growth model to calculate the new grain size is considered for three types of steel.

(15) $$D_{GG0}=D_{0}^{n}+k_{s}t\exp\left(\frac{Q_{GG}}{RT}\right).$$

The coefficients for Equation (15) are defined in Table 5.

#### 2.2.6. No-Recrystallization Temperature

It is known from the experiments that recrystallization may not occur every time, even though the conditions are met. This limitation comes from the temperature value. If it is below a certain value ($T_{nr}$), recrystallization will not take place. This temperature value is defined from the following equation given in [23]

(16) $$T_{nr}=A_{n}\varepsilon^{m}{\dot{\varepsilon }}^{n}t^{k},$$

where $A_{n}=905$, m=−0.045, n=−0.006, k=−0.024 and t is the time in seconds. These values are for high strength low alloy steel, experimentally obtained in [24]. The time in Equation (16) is accounted for only during the deformation process.

#### 2.2.7. Ferrite Grain Size Prediction

After the steel leaves the last rolling stand, it is cooled in a controlled way at the cooling bed. During the cooling process the austenite phase transforms into ferrite and perlite, especially for C-Mn steels. In this paper, only the ferrite transformation is considered, and the temperature at which the transformation occurs is called Ferrite Transformation Temperature (FTT). (FTT) for any type of steel in °C is calculated based on the following equation given in [25]:

(17) $$T_{FTT}=910-310\left[\%C\right]-80\left[\%Mn\right]-20\left[\%Cu\right]-15\left[\%Cr\right]-80\left[\%Mo\right]-\left[\%Ni\right].$$

The steel has to cool down until the maximum temperature goes below the FTT, and with that specific cooling rate at that temperature, the ferrite grain size can be predicted based on the following equation.

(18) $$D_{F}^{0}=\left(\alpha_{0}+\alpha_{1}C_{eq}\right)+\left(\alpha_{3}+\alpha_{4}C_{eq}\right){\dot{T}}^{-0.5}+\alpha_{4}\left(1-\exp\left(\alpha_{5}D_{A}\right)\right).$$

The necessary coefficients are defined in Table 6. It is important here not to ignore the residual strain ($\varepsilon_{r}$) which has an impact on reducing the ferrite grain size. If complete recrystallization occurred at each roll pass, the residual strains were obtained only from the last rolling stand. The following model by Sellars and Beynon [26] is considered in the simulations to calculate the final ferrite grain size.

(19) $$D_{F}=D_{F}^{0}\left(1-0.45\sqrt{\varepsilon_{r}}\right).$$

### 2.3. Coupling of Micro and Macro Models

Macro-scale simulation is followed by micro-scale simulation for each slice at a time except for the final ferrite grain size, which is calculated at the end. The major steps of the macro and micro-simulation steps and the coupling between them can be seen in Figure 5. The results were visualized for each slice, in particular at the exit from each rolling stand. The temperature, strain and strain rate fields are the inputs of the micro-scale model and were obtained from the macro-scale simulation results. This solution procedure represents a one way coupled system. When the simulation is completed, switching between or rearranging the micro-scale models and recalculating them is possible. Each micro-scale simulation took a few seconds. It was unnecessary to rerun the macro-scale simulation, which is time-consuming, to make a sensitivity study with different micro-scale model parameters. It was also possible to add entirely new micro-scale correlations into the database. There are two different ways of visualization of the micro-scale results. First is the type of recrystallization to see if it is partial or complete and when it happens. The second outcome is the grain size at each slice position, scaled from the minimum to the maximum.

## 3. Node Positioning

After the continuous casting in a typical steel production line, the strand proceeds to a reheating furnace to remove the cast dendritic structures and homogenize most alloying elements [2]. The strand afterwards deforms in the reversing rolling mill to obtain the desired pre-shape for the continuous rolling mill. The initial rectangular shape for the finishing rolling was not a perfect rectangle, but it had curved corners. We obtained the proper initial shape for use in the finishing rolling by using the reversing rolling mill simulation results. The regular node distribution was straightforward to generate and effective for the perfect rectangular shape. However, the regularly distributed nodes did not correctly provide the appropriate results near the curved edges.

Most of the rolling schedules used in the simulations may be categorized into two groups when rolling the billets. In the first group, oval grooves were used to achieve more oval or round-shaped forms. In the second group, primarily flat rolls were used to get more slab-like shapes. Regular node distribution was adequate for most of the slab simulations; however, regular nodes faced stability issues for a schedule that turns a rectangular billet into a round bar. Previously we used an Elliptic Node Generation (ENG) algorithm [27,28] for positioning the nodes in the domains with curved boundaries. This algorithm successfully maps a rectangular shape to an arbitrary shape with four sides and ensures high orthogonality. The downside of this method represents a mapped corner. This corner is potentially most tricky during any interpolation, just like in a rectangular domain. However, if we use an irregular node arrangement, this corner complexity will be lost completely. This provides a much more extensive deformation range with stable results.

### 3.1. Regular vs. Irregular Node Positioning

A fast, irregular node generation algorithm [29] is numerically implemented in the rolling simulation system. Additionally, the first row of internal points, just next to the boundaries, is aligned in the opposite direction of the unit normal of the nearest boundary point.

### 3.2. Node Repelling Algorithm

Due to the different texture of internal points near the boundaries and the rest of the internal points, an iterative node adjustment algorithm was used. In this way, the neighbouring nodes have a more uniform distance to the central nodes. The adjustment is based on a node repelling algorithm. The vector component of displacement $\Delta u_{i}$, due to repelling, is defined as

(20) $$\Delta u_{i}=C\sum_{j=1}^{N_{\max}}s\frac{p_{i}-p_{i}^{j}}{{\left(\|p_{i}-p_{i}^{j}\|\right)}^{n}}.$$

C is a smoothing factor usually taken between 0 and 1. The higher the node density, the smaller it should be. In this paper it is taken as (1/6). s is also a similar factor taken as 1, and reduced to 0.8 when at least one neighbouring point is located at a boundary. $N_{MAX}$ is a maximum number of the nearest nodes to consider without the boundary nodes. It was taken as 45 during the first iteration, and it was slowly reduced in each iteration until it reached seven nodes inside an influence domain.

## 4. Numerical Results

In this section, multiple numerical results from the represented multiscale rolling simulation system (Institute of metals and technology, version 7.6.4, Ljubljana, Slovenia) are illustrated. Macro-scale results are compared with regular and irregular collocation nodes and also compared with Finite Element Method (FEM) results. Later, an actual rolling schedule which consists of eight rolling stands, is applied. Micro-scale results are demonstrated in terms of grain size and recrystallization throughout the rolling schedule. The necessary values and definitions are given in Table 7.

### 4.1. Regular vs. Irregular Node Positioning—Slab

**Figure 6:**
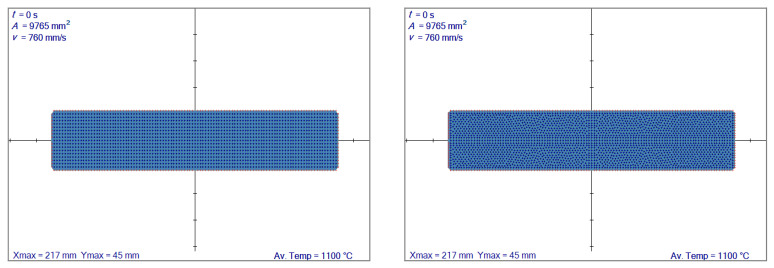
Comparison of different initial node arrangements. Regular node distribution (**left**), irregular node distribution (**right**). Av. Temp. stands for average temperature.

An initial slab with 217 × 45 mm^2^ is flat-rolled into 34.5 mm height. The material is elastic with Young’s modulus 10^6^ Pa and Poisson’s ratio 0.35. A regular node distribution with 546 nodes and irregular with 571 nodes are used, as shown in Figure 6. The deformation is calculated in 11 steps in both cases, and the coefficient of friction is taken as 0.1.

**Figure 7:**
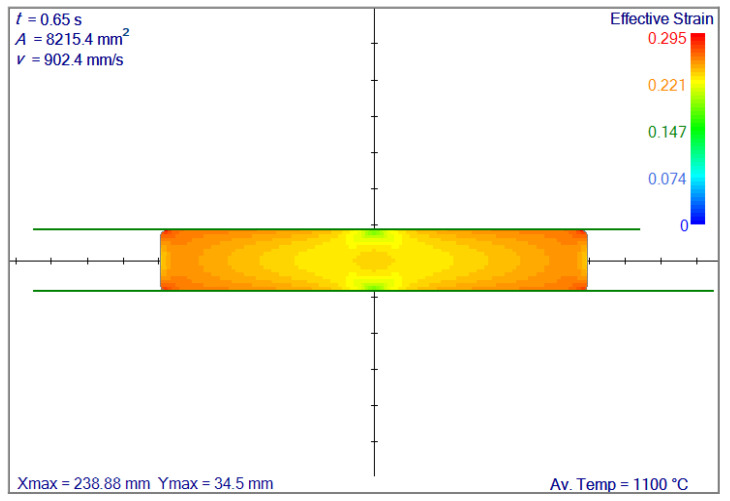

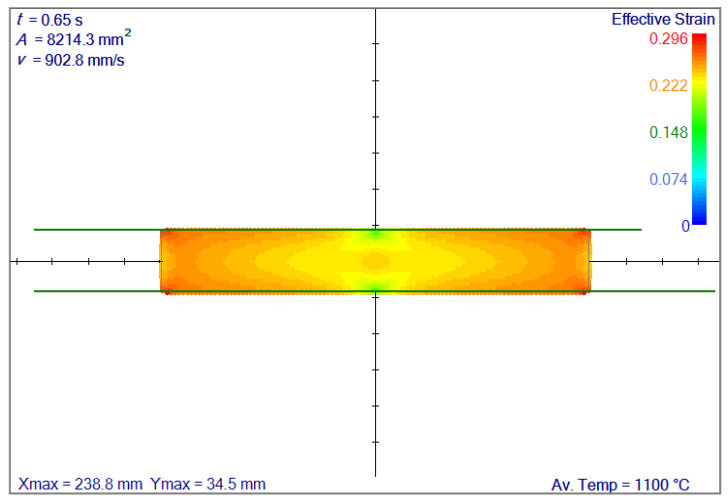
Comparison of effective strain fields after 10.5 mm of reduction. Av. Temp. stands for average temperature.

The results in terms of effective strain can be seen in Figure 7. The results are almost identical; therefore, for a slab that undergoes the flat rolling, irregular node generation has no distinct benefit.

### 4.2. Regular vs. Irregular Node Distribution—Real Shape

**Figure 8:**
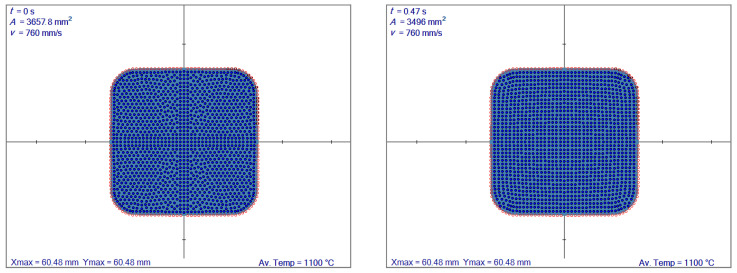
Irregular nodes (**left**) and regular nodes with mapping (**right**). Av. Temp. stands for average temperature.

More distinct differences occur when starting from an initial shape with curved corners, as obtained from the reversing rolling mill. The node arrangement is kept regular with ENG [28] and irregular with the algorithm [29], as shown in Figure 8. The regular and irregular node distributions involve 396 and 400 nodes, respectively.

**Figure 9:**
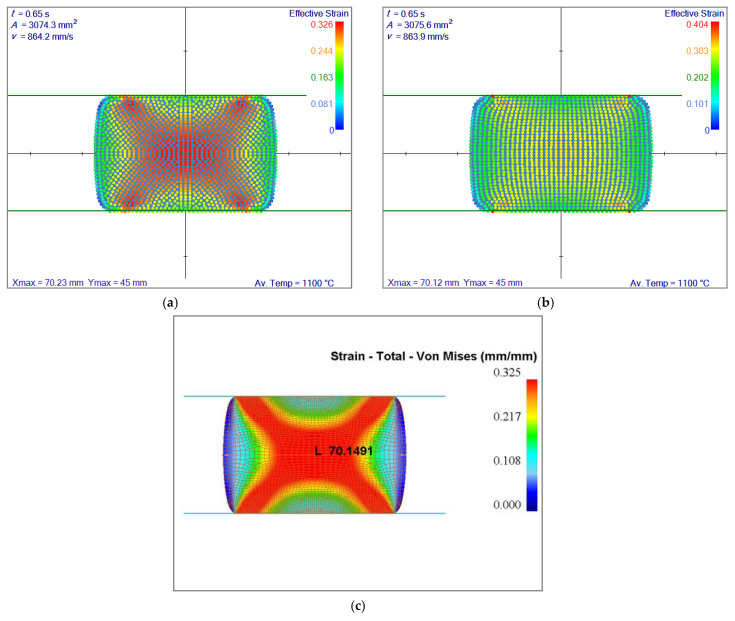
Comparison of simulated effective strain fields. Irregular nodes (**a**), regular nodes with mapping (**b**) and FEM results (**c**). The results of all three simulations are practically the same: (**a**) 70.23 mm, (**b**) 70.12 mm, (**c**) 70.15 mm. Av. Temp. stands for average temperature.

The instability issue at the edge of the first contact is still carried out until the end of the simulation in the regular node arrangement. In Figure 9, it is shown that irregular node arrangement made a better interpolation when the boundary shape diverges from regular (rectangular) geometry. Smoothed corners created problems with regular node arrangement. Comparing with FEM results in Figure 9c, the overshoot near the corner in Figure 9b is very clear. However, this can be overcome by using an irregular node arrangement where the corner information is completely lost as shown in Figure 9a.

### 4.3. Irregular Meshless Node Distribution vs. FEM

**Figure 10:**
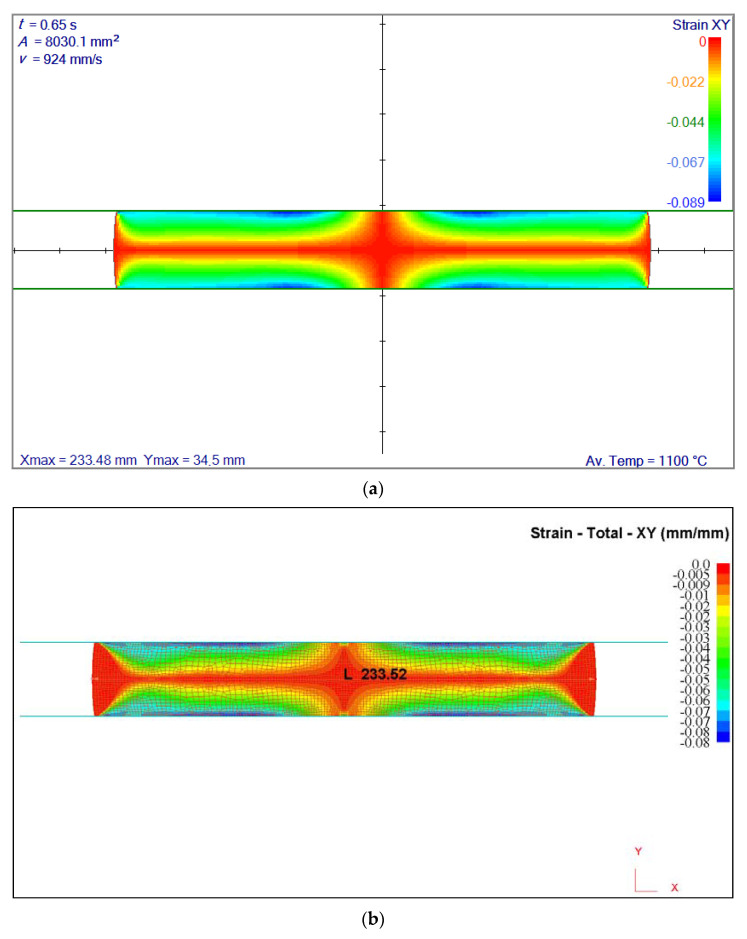
Comparison of strain fields. Meshless solution with irregular node arrangement is on top, FEM solution is shown at the bottom. The results of both calculations are almost the same: (**a**) 233.52 mm, (**b**) 233.48 mm. Av. Temp. stands for average temperature.

The same flat rolling example as in Section 4.1 is chosen here with an ideal plastic material model. Effective stress–effective strain relation is $\bar{\sigma }=589{\bar{\varepsilon }}^{0.2}$. A comparison is made with FEM code [30], and a good agreement is found. An example of a shear strain comparison is given in Figure 10 below. As expected, a good agreement has been found between the meshless results with irregular node arrangement and FEM.

### 4.4. Rolling Schedule with Eight Rolling Stands

**Figure 11:**
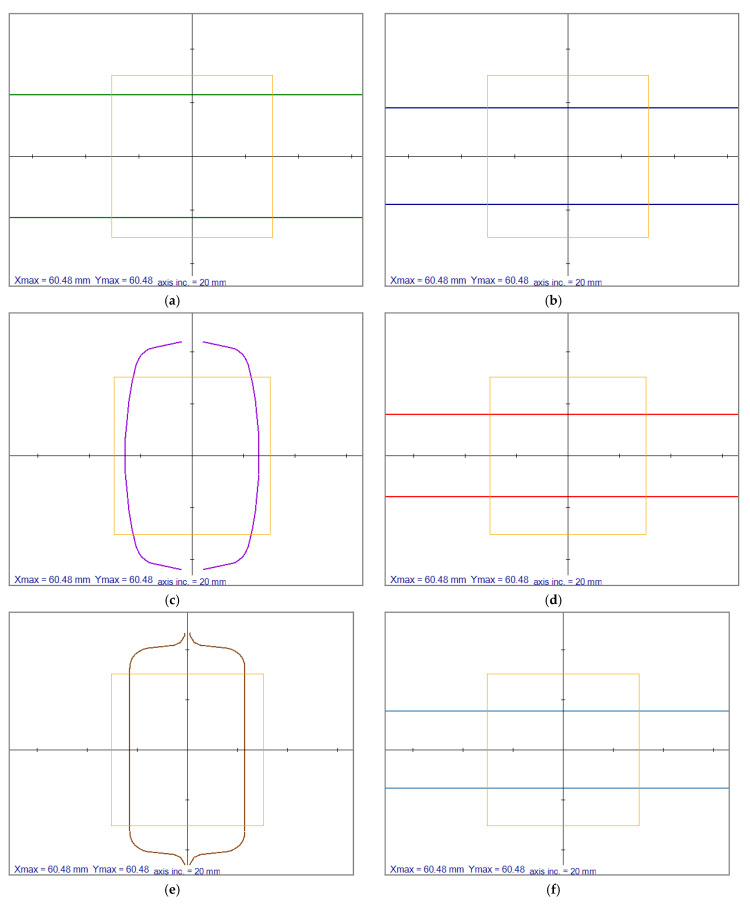

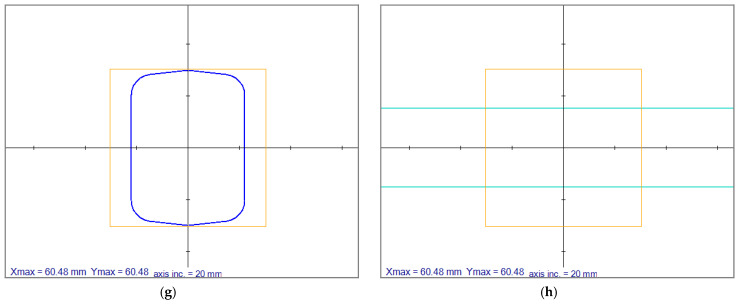
Demonstration of the roll shapes of each of the roll passes of an 8-roll rolling schedule. (**a**) first roll pass, (**b**) second roll pass, (**c**) third roll pass, (**d**) forth roll pass, (**e**) fifth roll pass, (**f**) sixth roll pass, (**g**) seventh roll pass, (**h**) eighth roll pass. Axis inc. is the length of axis increments.

In this section, a rolling schedule from the industry with specific grooves are shown in Figure 11 The same was used in all subsequent simulations. The initial size of the billet was 60.48 by 60.48 mm, where the corners are rounded with a radius of 11 mm. An irregular node distribution, the same as demonstrated in Section 4.2, is used. This rolling schedule aimed to obtain a 45 × 30 mm^2^ end shape within a half millimetre error range.

#### 4.4.1. Effective Strain Results

**Figure 12:**
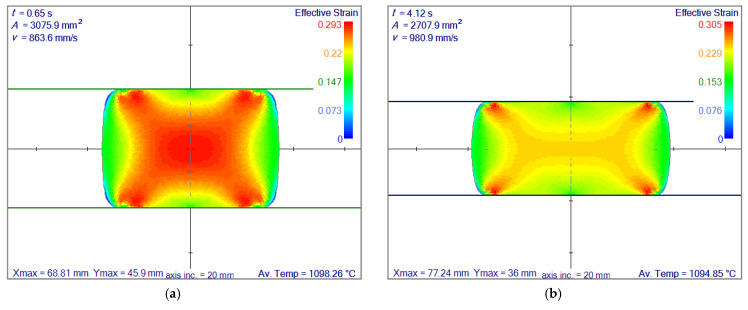

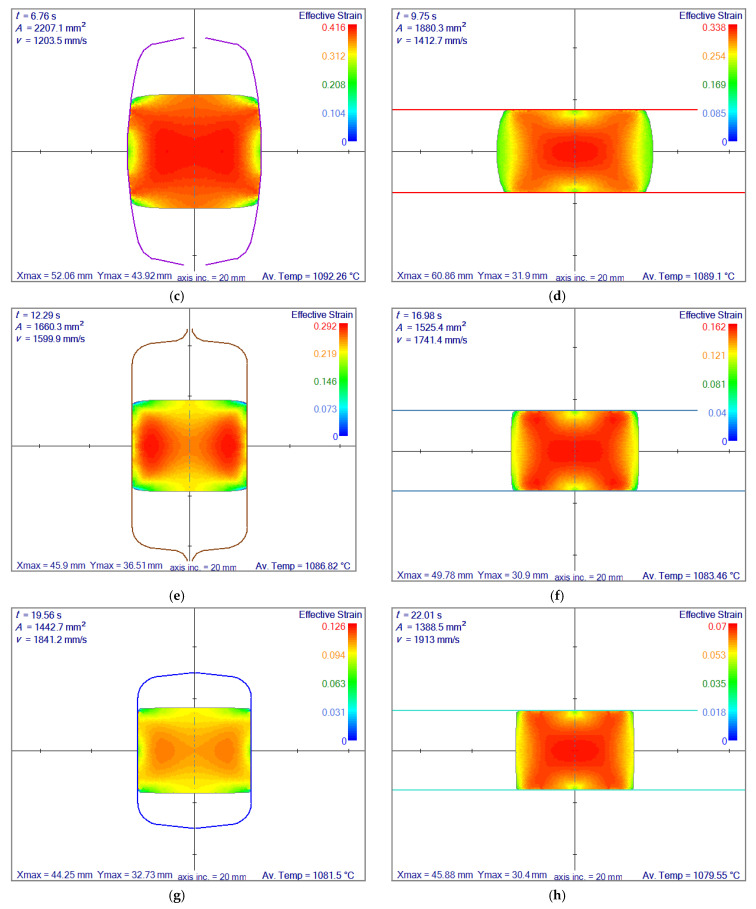
Simulation results of effective strain at the exit of each roll pass. (**a**) at first roll pass, (**b**) at second roll pass, (**c**) at third roll pass, (**d**) at forth roll pass, (**e**) at fifth roll pass, (**f**) at sixth roll pass, (**g**) at seventh roll pass, (**h**) at eighth roll pass. Axis inc. is the length of axis increments. Av. Temp. stands for average temperature.

Effective strain and temperature fields are the most critical inputs of the micro-scale models. The corresponding position and time of each slice have to be known throughout the simulation. Figure 12 represents the effective strain result of slices positioned at the exit of each roll pass. These results have shown the total effective strain through each of the rolling passes. Therefore these fields contain the highest possible values for each pass. These results are also crucial since they have a significant impact on the micro-scale simulation.

#### 4.4.2. Recrystallization Results at Each Pass

**Figure 13:**
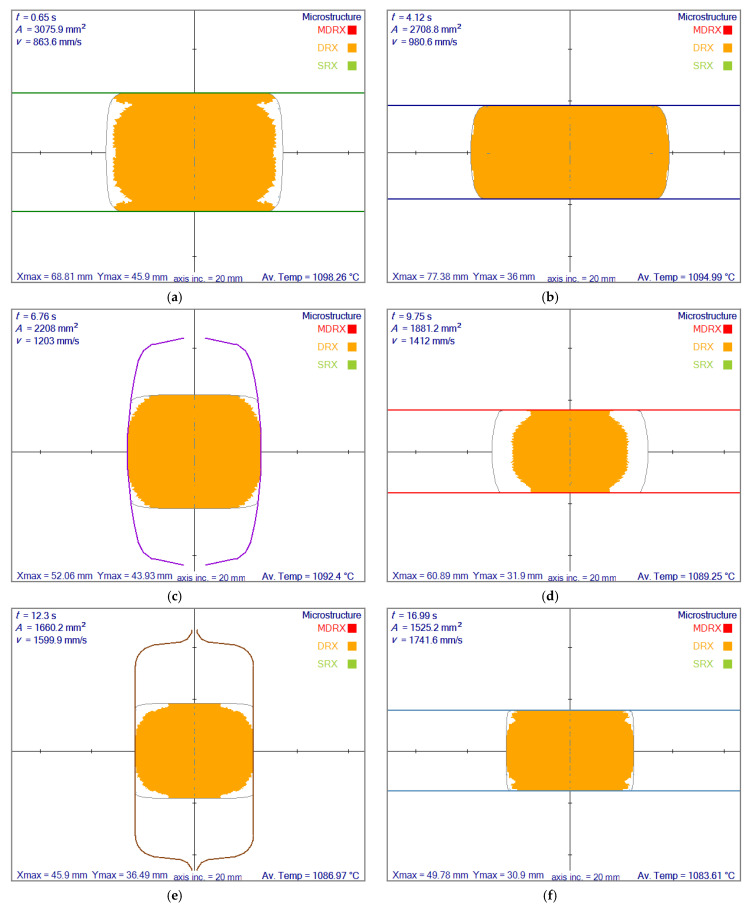

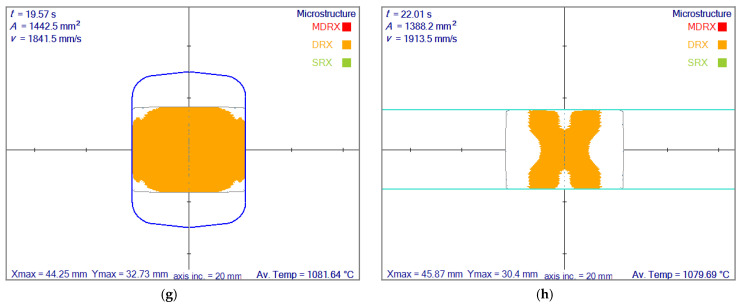
Recrystallization based on the model given by Yada [12] at the exit of each roll pass. (**a**) at first roll pass, (**b**) at second roll pass, (**c**) at third roll pass, (**d**) at forth roll pass, (**e**) at fifth roll pass, (**f**) at sixth roll pass, (**g**) at seventh roll pass, (**h**) at eighth roll pass. Possible recrystallization types are dynamic (DRX), meta-dynamic (MDRX) and static (SRX). Axis inc. is the length of axis increments and Av. Temps stands for average temperature.

In the simulations, the deformation occurred so rapidly that the time for meta-dynamic or static recrystallization was never reached. However, at each pass, soon after the first contact, a complete dynamic recrystallization was always achieved except for the model developed by Yada [12]. In that model, the dynamic recrystallization was partial at each pass. To contrast, the results obtained by Yada’s model are shown in Figure 13. In the three other models, there was always complete dynamic recrystallization.

#### 4.4.3. Recrystallization Results at Each Pass with Yada Model

The results of partial dynamic recrystallization obtained by the rolling simulation system are shown in Figure 13 for slices at each roll pass’s exit.

#### 4.4.4. Grain Size Comparisons of Different Models

**Figure 14:**
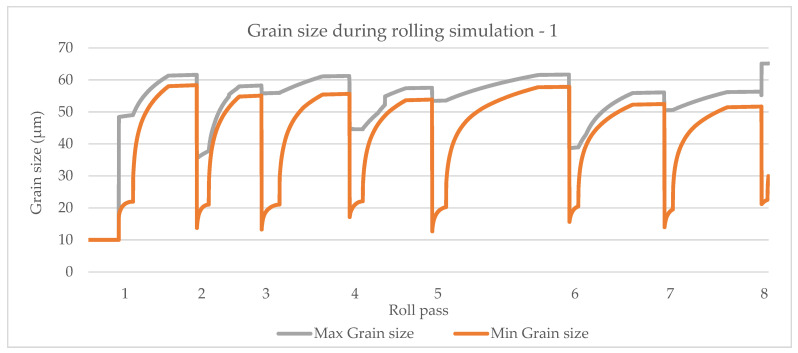
Simulation of maximum and minimum grain size based on the model by Sellars and Whiteman [14].

A rolling schedule is used as described in Section 4.4 and Figure 11. Grain size simulation results based on the model by Sellars and Whiteman [14] is shown in Figure 14.

**Figure 15:**
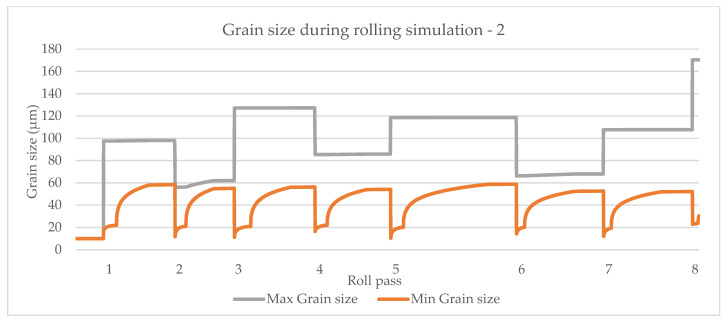
Simulation of maximum and minimum grain size based on the model by Yada [12].

**Figure 16:**
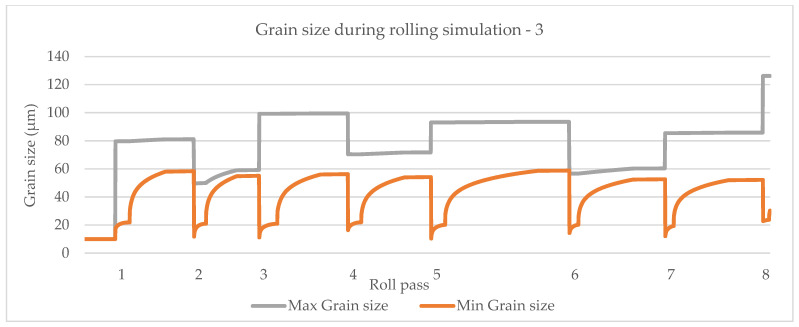
Simulation of maximum and minimum grain size based on the model by Manohar et al. [15].

**Figure 17:**
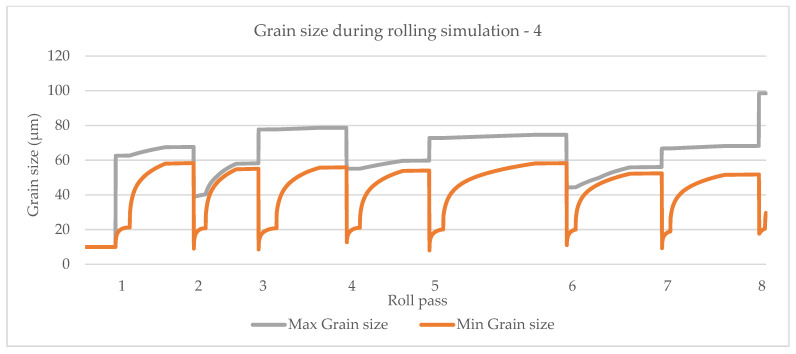
Simulation of maximum and minimum grain size based on the model by Hodgson and Gibbs [10].

The model by Yada [12] is shown in Figure 15, the model by Manohar, et al. [15] is shown in Figure 16 and the model by Hodgson and Gibbs [10] is shown in Figure 17. The grain growth model is obtained in [10]. In micro-scale simulations, each collocation point in a computational domain represents a small area with a uniform grain structure.

#### 4.4.5. Ferrite Grain Size for 16MnCr5

**Figure 18:**
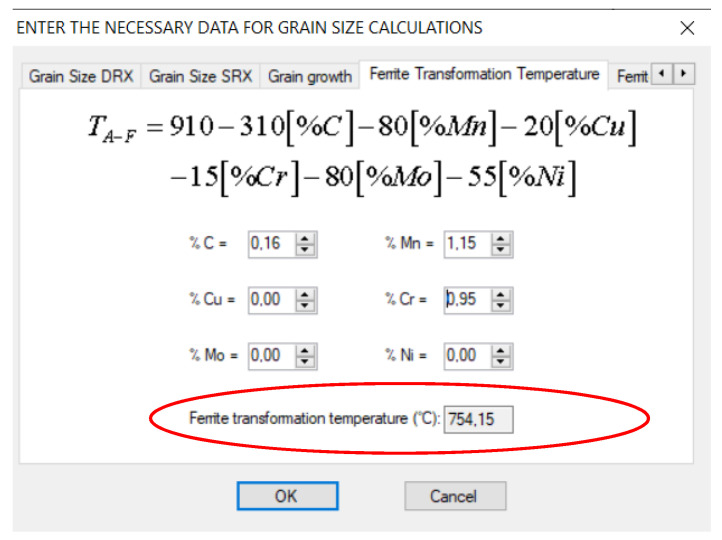
Ferrite transformation temperature used in the simulations based on the chemical composition of the steel.

**Figure 19:**
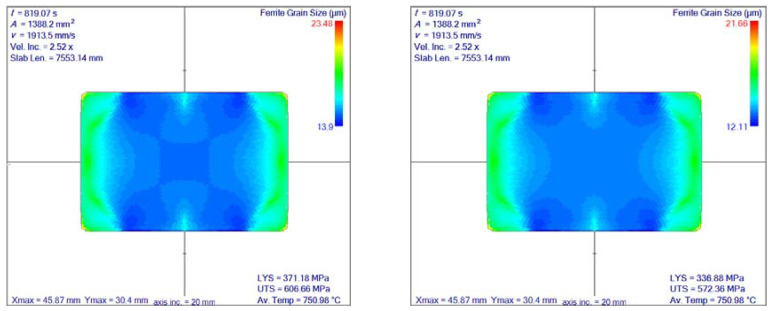
Comparison of final ferrite grain size based on the models given by Sellars and Whiteman (**left**) and Hodgson and Gibbs (**right**). Axis inc. is the length of the axis increments. Av. Temp. stands for average temperature. Vel. Inc. is the increase of the slab’s velocity towards the rolling direction. Slab. Len. Is the length of the rolled slab. LYS is the lower yields stress and UTS is the ultimate tensile strength.

For the calculation of the final grain size in the ferrite phase, 16MnCr5 steel is considered. This steel grade has 0.16% carbon, 1.15% manganese and 0.95% chromium. The ferrite transformation temperature is based on the model given by Hodgson and Gibbs, calculated as 754.15 °C, and the user interface is displayed in Figure 18. The real-time cooling to below FTT took 14 min; however, the computational time took twice as long. The final ferrite grain size and comparison between the two models is shown in Figure 19. A strong dependence on residual strain on the ferrite grain size is observed.

#### 4.4.6. No Recrystallization Temperature

**Figure 20:**
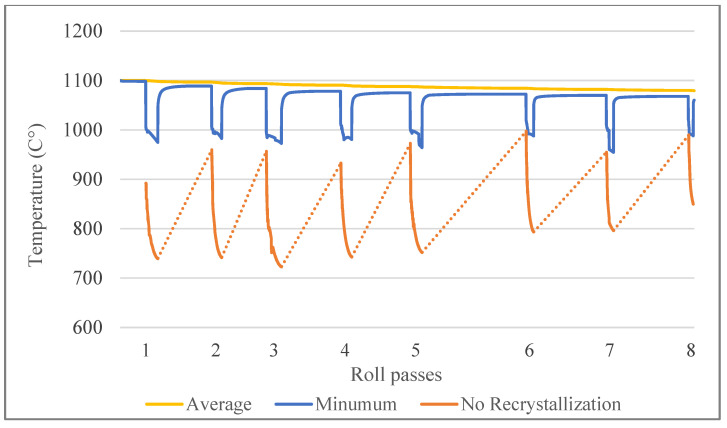
Comparison of estimated no-recrystallization temperature values at each rolling pass with the average and the minimum temperature values.

Based on the model expressed by Yang, et al. [24], no-recrystallization temperatures are calculated and compared with minimum and average temperature values for each slice position through the rolling simulation. The result is shown in Figure 20 below, and it is clear that for hot rolling at 1100 °C, the temperature of steel was always above the no-recrystallization temperature.

The positions of roll passes which are used in Figure 14, Figure 15, Figure 16, Figure 17 and Figure 20 are given in Table 8 below. The origin of this coordinate (z=0) is considered to be the initial slice’s position.

## 5. Discussion

Micro-scale calculations are based on a multitude of empirical correlations obtained from the experiments in the last four decades. The simulation system is capable of checking for meta-dynamic, dynamic or static recrystallization. When recrystallization occurs, a new grain size is calculated. If there is a full recrystallization, grain growth will start. At the end of rolling, cooling is simulated with the thermal model until it reaches the ferrite transformation temperature. The simulation ends with ferrite grain size prediction. A comparison of different models based on maximum and minimum grain sizes is shown. The accuracy of those results are limited to the accuracy of the input data which is simulated by the macro-scale model and the accuracy of the empirical micro-scale models. It is known from the industrial user experience that the accuracy of the macro-scale results is highly satisfactory. Therefore, if the empirical models are based on enough experiments, the expected grain-related results should be between the maximum and minimum simulated values.

Another outcome of this research is to be able to come up with new grain size prediction models in the future. This is why all the definitions are implemented as user-defined functions. Numerous additional experimental results are needed to obtain this goal.

The simulation system is currently limited to austenite and ferrite grain size predictions and assumes only one phase at a time. This will be improved in the future with multiple initial phase definitions and phase transformation criteria. The micro-scale models are also limited to the adequacy of those coefficients used. All the necessary coefficients for each steel type may not be found. It is a long and complicated experimental process to define all those parameters by analyzing numerous grain size data obtained by either using electron microscope [31] or ultrasound [32].

## 6. Conclusions

In the present paper, a rolling simulation system capable of predicting the grain structure is described. First, a macroscopic thermo-mechanical simulation is performed, based on a 2D solution over each cross-sectional slice. Displacement, strain, stress and temperature fields are calculated considering the defined process parameters (rolling schedule), heat transfer and material properties. The solution is achieved through a truly meshless radial basis function collocation method. A new irregular node generation algorithm is used, which is shown, by FEM comparison, to perform better on irregular domains. This method requires only the distribution of collocation nodes over a domain, without classical meshing. It is possible to represent the complex domain shapes more accurately with the irregular node distribution as with the regular node distribution. Due to the high possibility of recrystallization at each pass, strain filed during the last rolling stand leads to relatively smaller ferrite grain size. It was shown in the simulations that dynamic recrystallization always took place at each roll pass. Meta-dynamic and static recrystallizations did not occur due to time restrictions. It was also expressed that no-recrystallization temperatures most probably does not stop recrystallization during hot rolling of steel above 1100 °C. A list of all the correlations used in this paper may be found in Appendix A.

## Figures and Tables

**Table 1:** Parameters used in the critical strain definition for carbon–manganese steel in [11,12,14] and low carbon steel in [13].

Coefficients	[11]	[12]	[13]	[14]
$A_{c}$	6.82 × 10^−4^	4.76 × 10^−4^	4.9 × 10^−4^	4.9 × 10^−4^
$Q_{d}$	312,000	66,500	300,000	312,000
p	0.13	0	0.17	0.15
q	0	0	0.3	1

**Table 2:** Parameters used in the grain size equation for dynamic recrystallization of carbon–manganese steel.

Coefficients	[10]	[12]	[14]	[16]
$A_{d}$	16,000	22,600	1800	16,000
r	−0.23	−0.27	−0.15	−0.23
$Q_{d}$	312,000	267,100	312,000	300,000

**Table 3:** Parameters used in the grain size equation for meta-dynamic recrystallization of carbon–manganese steel [10,14,16], and low carbon steel [10].

Coefficients	[10]	[13]	[14]	[16]
$A_{m}$	26,000	25,000	1600	26,000
r	−0.23	−0.23	−0.11	−0.23
$Q_{d}$	312,000	312,000	312,000	300,000

**Table 4:** Parameters used in the grain size prediction model when static recrystallization takes place. These parameters are obtained by Zhang [16] for high carbon steel and Sellars [8] for carbon manganese steel.

Coefficients	[13]	[7]
$A_{s}$	0.5	1
m	−0.67	−0.5
r	0.67	0.4

**Table 5:** Parameters used in the grain growth calculation when complete recrystallization takes place. These parameters were obtained by Hodgson and Gibbs [10] for three types of steel.

Coefficients	C-Mn-V	C-Mn-Ti	C-Mn-Nb
n	7	10	4.5
$k_{s}$	1.45 × 10^27^	2.6 × 10^28^	4.1 × 10^28^
$Q_{GG}$	−400	−437	−435

**Table 6:** Parameters used to calculate ferrite grain size for C-Mn steel.

Coefficients	[10] (%C) + (%Mn)/6 > 0.35	[10] (%C) + (%Mn)/6 < 0.35	[26] C-Mn-Ti
$\alpha_{0}$	−0.4	22.6	1.4
$\alpha_{1}$	6.37	−57	0
$\alpha_{2}$	24.2	3	5
$\alpha_{3}$	−59	0	0
$\alpha_{4}$	22	22	22
$\alpha_{5}$	0.015	0.015	0.015

**Table 7:** Parameters used in the rolling simulation.

**General Data**	
Initial size of the slab	60.48 × 60.48 mm
Initial temperature field of the slab	1100 °C
Entry velocity towards the rolling direction	760 mm/s
Coefficient of friction	0.15
Initial grain size	10 µm
**Material Model**	
$\bar{\sigma }\left(\bar{\varepsilon },\dot{\bar{\varepsilon }},T\right)=589{\bar{\varepsilon }}^{0.214}{\dot{\bar{\varepsilon }}}^{0.2}\exp\left(\frac{38,000}{RT}\right)\;\mathrm{MPa},$ for 16MnCr5 alloyed steel	
Thermal Model	
Thermal conductivity	29 W/mmK
Specific heat	630 J/KgK
Density	7450 kg/m^3^
Heat transfer coefficient to air	20 W/m^2^K
Heat transfer coefficient to roll	10,000 W/m^2^K
Roll surface temperature	600 °C
Groove and Roll Data	
Roll radii	230 mm
Roll gaps	34.5–26.5–118–22.5–112–20–107.4–18.2 (mm)
Groove geometries (H-horizontal, V-vertical)	flat (H)—flat (H)—oval box (V)—flat (H)—flat box (V)—flat (H)—flat box (V)—flat (H)
Distance between rolling stands	3 m (for the first five rolling stands), 4.5 m (after the fifth rolling stand).

**Table 8:** Positions of centres of each roll along the rolling direction with reference to the initial slice position.

Passes	1	2	3	4	5	6	7	8
Position on rolling direction (mm)	500	3500	6100	9700	13,300	20,800	25,300	29,800

## Data Availability

Data sharing not applicable.

## Appendix A

		**C-Mn**				**Low C**	**High C**	**C-Mn-V**	**C-Mn-Ti**	**C-Mn-Nb**	**HSLA**
Critical strain	A	6.82 × 10^−4^	4.76 × 10^−4^		4.9 × 10^−4^	4.9 × 10^−4^					
	$Q_{d}$	312,000	66,500		312,000	300,000					
	p	0.13	0		0.15	0.17					
	q	0	0		1	0.3					
Dynamic recrystallization	A	16,000	22,600	1800	16,000						
	r	−0.23	−0.27	−0.15	−0.23						
	$Q_{d}$	312,000	267,100	312,000	300,000						
Meta-dynamic recrystallization	A	26,000		1600	26,000	25,000					
	r	−0.23		−0.11	−0.23	−0.23					
	$Q_{d}$	312,000		312,000	300,000	312,000					
Static recrystallization	A	1					0.5				
	m	−0.5					−0.67				
	r	0.4					0.67				
Grain growth	m							7	10	4.5	
	$k_{s}$							1.45 × 10^27^	2.6 × 10^28^	4.1 × 10^28^	
	$Q_{GG}$							−400	−437	−435	
No-recrystallization temperature	A										905
	m										−0.045
	n										−0.006
	k										0.024
Ferrite grain size	$\alpha_{0}$	−0.4	22.6						1.4		
	$\alpha_{1}$	6.37	−57						0		
	$\alpha_{2}$	24.2	3						5		
	$\alpha_{3}$	−59	0						0		
	$\alpha_{4}$	22	22						22		
